# Health and Social Care Outcomes in the Community: Review of Religious Considerations in Interventions with Muslim-Minorities in Australia, Canada, UK, and the USA

**DOI:** 10.1007/s10943-022-01679-2

**Published:** 2022-10-01

**Authors:** Helen McLaren, Mohammad Hamiduzzaman, Emi Patmisari, Michelle Jones, Renae Taylor

**Affiliations:** 1 College of Education, Psychology and Social Work, Flinders University, GPO Box 2100, Adelaide, SA 5001 Australia; 2 Faculty of Health, Southern Cross University, Gold Coast, Australia; 3 Community Development, Education & Social Support Australia (CDESSA) Inc., Adelaide, Australia

**Keywords:** Muslim, Minority, Health, Social care, Community, Integrative review

## Abstract

The aims of this integrative review included examining the intervention characteristics, religious tailoring, and behavioural outcomes of health and social care interventions with Muslim-minorities in Australia, Canada, UK, and the USA. Nineteen articles were included, and each showed some level of improved health and social care outcomes associated with interventions that were religiously tailored to Islamic teachings, and when notions of health were extended to physical, psychological, spiritual and social domains. Future studies should measure levels of religiosity to understand whether religiously tailored interventions produce a significant intervention effect when compared to non-religiously tailored interventions with Muslims.

**Supplementary Information:**

The online version contains supplementary material available at 10.1007/s10943-022-01679-2.

## Introduction

Indifference with the ethnoreligious needs of Muslim-minorities has led to a complex array of poorly understood barriers to health and social care (Hanrieder, 2017), affecting Muslims’ wellbeing (Patmisari et al., 2022). Mainstream indifference leads to marginalization and, consequently, low levels of health literacy (Hamiduzzaman et al., 2022; Shahin et al., 2021), inequitable access to community services and care (Ishaq et al., 2021; Samari, 2016), and poorer health outcomes (Shahin et al., 2021; Shlala & Jayaweera, 2016). Marginality is a key factor that hinders the confidence of Muslim-minorities in their access of mainstream health and social care. In our recent study of multicultural quality of life predictive effects of wellbeing among an Australian sample, feeling safe and having access to religiously appropriate resources was seen as crucial to the health of this Muslim community (Patmisari et al., 2022). Nonetheless, the health and social care of this community was equally impacted by the persistence of anti-Muslim sentiment and ethnoreligious misunderstanding across nations and time (Elkassem et al., 2018; McLaren & Patil, 2016; Patil & McLaren, 2019).

In countries where Muslims are minority, there may be few policies in health and social care settings, or motivation, to either tackle Islamophobia or to operationalize ethnoreligious components into care (Allen, 2021; Griera & Martínez-Ariño, 2016; Silva et al., 2018). For example, Bawadi et al. (2020) studied the experiences of Arab Muslim women in maternity hospitals in the UK and found discrimination and prejudice, emotional coldness from staff, and ideological conflicts towards the women during childbirth and aftercare. African Muslim women hospitalized in Spain likewise experienced religious discrimination when denied support to worship or pray during their confinement (Plaza del Pino et al., 2020). The absence of religious tailoring of policy and practice in these examples represents both disrespect and neglect in the wholistic care of Muslims, and denial of basic human rights. Abuelezam et al. (2018) noted in their review of USA health services that religious information was collected on intake, but the information was not used to inform the health, caring or decision-making with Muslims populations. Alternatively, McLaren et al. (2021) in their review of religiosity in health and wellbeing interventions with Muslim-minorities, showed that praying, fasting and meditating *(dhikr)* was important to Muslims receiving care. However, research interest in taking positive action towards accommodating Islamic beliefs in health and social care in the community settings of non-Muslim countries is relatively recent (i.e. Hamdiui et al., 2021; Ishaq et al., 2021; Saidun et al., 2019).

As a basis for generating baseline evidence in which to culturally tailor our own programme development and research with a local Muslim community in Australia, we undertook an integrative review of literature reporting health and social care outcomes with Muslim-minority communities. Our focus was on academic articles reporting research outcomes of interventions in Australia, Canada, UK, and USA. Since ‘interventions affect and are affected by both system and client characteristics producing desired outcomes’, the Quality of Health Outcomes Model (QHOM, Mitchell et al., 1998, p. 44) provided an analysis frame to deductively theme the dynamic interconnection between clients, systems, interventions, and outcomes across the studies reviewed.

We adopt the definition of religious tailoring by Worthington Jr et al. (2013) as interventions that accommodate one’s faith or tradition, spiritual practices, values, or beliefs. We note, however, that majority of literature on the provision of religiously accommodative interventions is with Christians. The current efficiency of religious tailoring or adaptations by service providers to ensure good health and social care outcomes with Muslim-minorities in Anglophone countries is not known. Adaptations are an important implementation strategy (Miller et al., 2020). Of interest here is the nature and scope of adaptations in association with outcomes reported.

## Methods

An integrative review was chosen to capture the heterogeneity of research, e.g. quantitative, qualitative, and mixed methods. Our initial intention was to include articles reporting Mosque-based social care and welfare programmes in countries where Muslims were minority. When our initial scope returned too few results, we expanded the eligibility criteria to include both health and social care interventions with Muslim-minorities in community settings.

Our systematic search followed PRISMA guidelines (Page et al., 2021), with Covidence (2015) used to facilitate the screening based on our inclusion and exclusion criteria. Studies were deductively coded and arranged into themes (Erlingsson & Brysiewicz, 2017). Included articles were evaluated for risk of bias using the mixed methods appraisal tool (MMAT) (Hong et al., 2018). The QHOM offered a framework to identify associations between religious tailoring of interventions and the health and social care outcomes of Muslim-minorities in non-Muslim countries.

We included studies reporting on Muslim-minorities, and sub-samples of Muslims. Originally returning 20 studies, one from Austria was excluded (Bader et al., 2006), enabling a discrete focus on Anglophone countries (Australia, Canada, UK, and USA). Peer-reviewed academic journals in English language reporting primary research were included in the current review. Review and opinion articles, conference papers and theses, and grey literature were excluded to prioritize academic rigour in the sample.

### Search Strategy

A database search was completed in April 2021, followed by ancestry searching of reference lists and Google Scholar tracking of citing authors. There were five major databases in the electronic search: ProQuest, Scopus, Web of Science, PsycINFO, and Informit. Searches likely to capture articles on health, social and welfare interventions with Muslim-minorities included terminologies such as: ‘Muslim’, ‘Islam’, ‘Mosque’, ‘minority’, ‘health’, ‘wellbeing’, ‘community’, and specific types of interventions likely in community health and social care (Table S1, databases and search syntax). No date, country of author or publication country thresholds were applied to the search.

**Fig. 1:**
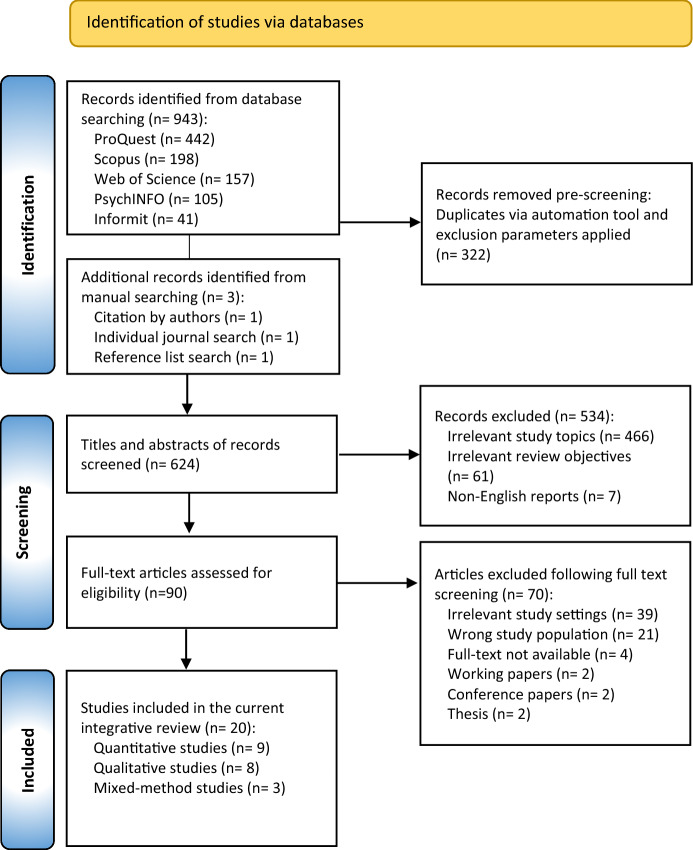
PRISMA flow diagram of articles included

The initial search returned 936 items. Duplicates (n = 322) were removed, leaving 625 items for titles and abstract screening (completed by two authors). Full-text screening of 90 items resulted in 19 eligible articles, inclusive of three identified from citation tracking of included articles and excluded review studies. The Prisma Flow diagram provides a visual overview of the systematic search and inclusion/exclusion process (Fig. 1).

Quality assessments were completed using the MMAT tool by Hong et al. (2018), which considers clarity of research questions, rationale, study design, and strength of analysis (Table 1). All articles contained a well-defined research question and collected data appropriate to answer. Two of the four mixed-methods studies and three qualitative studies did not provide justification of research design. Three qualitative studies did not clearly articulate data collection processes and coherence between qualitative data sources, collection, analysis, and interpretation. Non-response bias, missing data and face validity in quantitative studies were provided as reason for non-participation, and risk of bias associated with survey instruments were generally not discussed.

**Table 1:** The quality appraisal for the selected studies using MMAT tool

Mixed methods studies	Are there clear research questions?	Do the collected data allow to address the research questions?	Is there an adequate rationale for using a mixed methods design to address the research question?	Are the different components of the study effectively integrated to answer the research question?	Are the outputs of the integration of qualitative and quantitative components adequately interpreted?	Are divergences and inconsistencies between quantitative and qualitative results adequately addressed?	Do the different components of the study adhere to the quality criteria of each tradition of the methods involved?
Islam et al. (2012)	Y	Y	N	Y	Y	Y	Y
Zoellner et al. (2018)	Y	Y	N	Y	Y	Y	Y
Hassan et al. (2021)	Y	Y	N	Y	Y	Y	Y

Qualitative studies	Are there clear research questions?	Do the collected data allow to address the research questions?	Is the qualitative approach appropriate to answer the research question?	Are the qualitative data collection methods adequate to address the research question?	Are the findings adequately derived from the data?	Is the interpretation of results sufficiently substantiated by data?	Is there coherence between qualitative data sources, collection, analysis and interpretation?
Grace et al. (2008)	Y	Y	Y	Y	Y	Y	Y
Darko et al. (2020)	Y	Y	Y	Y	Y	Y	Y
Vu et al. (2018)	Y	Y	Y	Y	Y	Y	Y
Padela et al. (2018)	Y	Y	Y	Y	Y	Y	Y
Abdulwasi et al. (2018)	Y	Y	Probably Y	Probably Y	*C*	Y	Probably Y
Tse (2002)	Y	Y	C	C	Probably Y	Y	Y
Marinescu et al. (2013)	Y	Y	Probably Y	Probably Y	Y	Y	Probably Y

Quantitative randomized controlled trials	Are there clear research questions?	Do the collected data allow to address the research questions?	Is randomization appropriately performed?	Are the groups comparable at baseline?	Are there complete outcome data?	Are outcome assessors blinded to the intervention provided?	Did the participants adhere to the assigned intervention?
King et al. (2017)	Y	Y	Y	Y	Y	Probably Y	Y
Islam et al. (2018)	Y	Y	Probably Y	Y	Y	Y	Y

Quantitative non-randomized studies	Are there clear research questions?	Do the collected data allow to address the research questions?	Are the participants representative of the target population?	Are measurements appropriate regarding both the outcome and intervention (or exposure)?	Are there complete outcome data?	Are the confounders accounted for in the design and analysis?	During the study period, is the intervention administered (or exposure occurred) as intended?
Padela et al. (2019)	Y	Y	Y	Y	Y	Y	Y
Siddique and Mitchell (2013)	Y	Y	C	Y	Probably Y	Y	Y
Padela et al. (2018)	Y	Y	Y	Y	Y	Probably Y	Y
Chaudhary et al. (2019)	Y	Y	C	Y	Probably Y	Y	Y
Banerjee et al. (2017)	Y	Y	C	N	Y	Y	Y

Quantitative descriptive	Are there clear research questions?	Do the collected data allow to address the research questions?	Is the sampling strategy relevant to address the research question?	Is the sample representative of the target population?	Are the measurements appropriate?	Is the risk of nonresponse bias low?	Is the statistical analysis appropriate to answer the research question?
Bader, et al. (2006)	Y	Y	C	Y	Y	Y	Y
Padela et al. (2018)	Y	Y	C	C	Probably Y	Y	Y

Probably Y = Probably yes = few aspects of the questions are confirmed for some sleeted studies = -other nots, Y = yes; N = No and C = Can’t tell

### Synthesis and Analysis of Results

Study characteristics extracted from each article included authors, aims, population, interventions, measure/instruments, and reported intervention outcomes. The intention was to enable a broad overview of activities targeted at improving health and social care, patterns across the interventions, any ethnoreligious barriers that may have influenced participation by Muslim women and men, and intervention outcomes. Cross-checking and confirmation of information by the co-authors ensured trustworthiness in the review findings.

Data analysis of study interventions and outcomes used a combination of content and deductive analysis. Guided by methods established by Erlingsson and Brysiewicz (2017), extraction and analysis involved coding, developing categories and meanings, and condensed meaning units. Deductive thematic analysis guided by the QHOM framework enabled searching for three characteristics, or themes: the system, client, and intervention, to be considered in the context of a fourth theme: health and social care outcomes.

### Findings

Nineteen articles were reviewed. They were published between 2002 and 2021. There were eight quantitative studies (2 RCT, 5 non-RCT, and 1 quantitative descriptive), eight qualitative and three mixed-method studies. Four studies (2 qualitative and 2 quantitative non-randomized) were written by the same lead author (Padela et al., 2018; Padela, Malik, Ally Syeda, et al., 2018; Padela, Malik, Vu, et al., 2018; Padela et al., 2019; Table 2).

**Table 2:** Overview of studies in QHOM

Author/Study Aims	System Characteristics	Client Characteristics	Interventions	Outcome Measures/ Instruments	Impact of Interventions on Outcomes
Hassan et al. (2021) aimed to identify the effects of an Islamic centred educational program surrounding addictions for adult Muslims	A mosque-centred, psychoeducational intervention delivered by seven Muslim psychiatrists and psychiatry residents and targeted towards Adult Muslims in nine mosques in Toronto, Canada. Bilingual facilitators (Urdu and Arabic) were provided	93 adult (above 18 years old) Muslim participants, and fluent in English, and had an interest in addiction psychoeducation	A 90-min mosque-based seminars encompassing Islamic content from Quran and Hadith as well as scientific evidence related to addictions. The seminars were conducted in nine different mosques	Self-reported validated questionnaire (pre-and post- intervention) and post-intervention written questionnaire	There was a significant increase in the participants’ self-reported knowledge. A more positive attitude was observed. An increase was observed in motivation to seek help from a medical doctor and mental health professional post-seminar as compared to baseline
Darko et al. (2020) sought to assess the enactment of the Safer Ramadan initiative as part of diabetes prevention program	A mix of health care professionals (diabetes specialist nurses) and various experts (non-health professionals with specific facilitator skills) explored how to facilitate a program with the intention of it to be delivered by Muslim primary health. care workers in a community centre. The interventions were conducted in the cities of Leicester and Northampton in the East Midlands in England	80 Muslims participants (68 participants n Leicester, and 12 participants in Northampton	Group sessions promoting the ‘Safer Ramadan’ initiative were provided throughout two cities prior to Ramadan 2017	Qualitative interviews and focus groups	Low engagement of primary health practitioners to refer their clients for attending the ‘Safer Ramadan’ program. Successful high attendance was from a Bangladeshi community centre. All relevant stakeholders including, GPs, nurses, and imams have a role to raise public awareness and are active in referring patients to this program. Positive feedback on the content of the course especially about the nutrition guidelines. The facilitators had limited language skills to support non-English
Padela et al. (2019) planned to appraise a religious-sensitive program for breast cancer screenings among Muslim women	The program was developed by representatives from several research teams, social service associations, community centres, and community mosques. The program was specially tailored towards South Asian and Arab Muslim Women in Chicago, USA	58 female Muslims (South Asian, 29, and Arab, 18) without history of breast cancer and mammogram, fluent in English, aged between 40–74 years	A two-day religiously sensitive group session workshop to explore mammography hesitancy	Pre-and post-intervention questionnaires and 13 focus group discussions and 19 individual interviews	Analyses revealed a statistically significant increase in participants’ knowledge about mammography. Participants’ overall agreement with facilitator beliefs showed an ascending trend. There was a significant reduction in agreement with the belief "Breast Cancer Screening is not important because God decides who will get cancer"
Chaudhary et al. (2019) set out to determine the influence of psycho-educational healthcare training program for Syrian refugee mosque attendees	Medical doctors, nurses, and an on-site psychologist led the program targeted towards the local Syrian refugee community in Baltimore, USA. Handouts were provided both in English and Arabic	18 Muslim Syrian Refugees (one male), five spoke fluent Arabic, aged 21–68 years (General health issues) 47 community members including 14 Syrian refugees (mental health issues related to heart health) 52 community members including 22 Syrian refugees (vaccination and preventive care)	A 6-week program aimed at exploring health-related topics in the Syrian refugee communities. The classes were divided into two topics: health-related diseases (HD) and healthcare system (HS)	Questionnaires (including a free-response section), and interviews	The HD class scored higher in perceived objectives achievement than HS class. There were no statistically significant differences for other questions between the two classes. Participants wanted to be informed about mental health in particular related to domestic violence, and children. A significant number of Syrian refugees attended two community awareness programs (“Heart health awareness” and “Vaccination and preventive care”). Following the intervention success, a part-time case manager was hired through the mosque for follow-ups and to continue the service for Syrian refugee
Zoellner et al. (2018) evaluated a culturally sensitive program in community mosques for Muslim refugees who had experienced trauma	The mosque-based intervention was led by a research team, Imams, and leaders of the Somali health board and local Somali associations. The target population consisted of Somali Muslim refugees in a large city in the USA	Study 1: Assessment, 39 Somali Muslim refugees (25 Somali men and 15 Somali women) aged above 18 years (asking specific age was considered insensitive by Somalis) Study 2: Pilot study. One group of three women leaders, one group of six men leaders, conducted in English	Study 1: Conducted to assess PTSD and community interests in “Islamic Trauma Healing” program Study 2: “Islamic Trauma Healing” pilot program delivered via two 4-h sessions by same sex facilitators as group participants	Study 1: An anonymous survey, including the Primary Care PTSD Screen for DSM-5 (written in English and Somali) Study 2: Pre–post-measures to assess PTSD severity, wellbeing, and participants’ satisfaction on the program; followed by focus group discussion	Approximately 23% participants experienced PTSD. A strong perceived need and match with the Islamic faith for the intervention, with large effects from the pre- to post-group. High satisfactory with the program was reported by participants. There was a significant decrease in PTSD severity and symptoms. Qualitative data confirmed that the program was well-received by the participants. Participants valued community connection and Islamic content the most
Vu et al. (2018) assessed the attitudes of Muslim women towards health promotion interventions within mosque-type settings	This mosque-based study did not mention mixed professionals’ involvement. The investigators explored Muslim women’s views in order to deliver effective health messaging in Chicago, USA	19 female Muslims from African American, South Asian, Arab, and Southeast Asian ethnicities, aged 40–74 years, have a primary care physician, mostly Sunni affiliated, religiosity rated 5 (somewhat religious)-10 (very religious)	Exploring the value of delivering health education and interventions by Imams in Friday sermons within mosque-based health initiatives	A focus group discussion	Around 84% of women accepted Imam-led sermon on women’s health, whereas 16% of them disapproved male Imams preach about women’s issues. Friday sermons were perceived appropriate as health education modality by less than 50% of women. As looking after own health is part of Muslims’ religious duties, when delivering the sermons, women’s health needs to be framed in accordance with Islamic principles
Padela, Malik, Vu, et al. (2018) sought to design and deliver a theoretical framework for communicating religious sensitive health promotion messages	This religiously tailored initiative was led by various researchers and experts focussing on Muslim-American women from various community groups, in USA	240 American Muslims aged above 40 years	Program design which incorporated the belief systems of participants were extracted from three different stages Stage 1 identified breast cancer screening rates and how religious traits were correlated amongst Muslim women in Chicago Stage 2 explained beliefs and behaviours surrounding breast cancer screenings and the role religion plays in forming these beliefs Stage 3 prompted opinions about mosque-based program strategies	A 3R-reframing, reprioritizing, and reforming model	Established a theoretical model to convert behavioural concepts into actionable procedures for creating religiously customized health information, comprising three methods: reforming, reprioritizing, and reframing. Religious concepts were applied to overcome belief barriers. Religious ideas and principles were then drawn upon to modify alternate information about living a healthy daily life Informal constructive criticism from those devising and delivering the information indicates that the framework was beneficial, while initial feedback suggests that the information and syllabus was in line with their values
Padela, Malik, Ally Syeda, et al. (2018) detailed the results of a community health program targeted at increasing breast cancer screening intentions among Muslim women	The mosque-based intervention format focussed on peer discussions and expert psychoeducation targeted at Muslim American women, in USA	58 female American-Muslims (South Asian, Arab), age (40 – 70 years), fluent in English, and no breast cancer screening within the last two years	A two-class initiative aimed at increasing mammography engagement by providing Islamic sensitive health messaging	Pre- and post-intervention data was gathered from participants self-filled surveys. Breast cancer screening at 6 months and 1 year following intervention	Analyses revealed a statistically significant rise in perceived probability to engage in breast cancer screenings. This rise was continued at 6 months amongst the 38 women who underwent mammography within 12 months of the program. A regression analysis revealed that marriage was positively correlated with behaviour change
Padela et al. (2018) explored the viability of health campaigns via the use of sermons within American mosques	Mosque-based intervention: two different mosque locations in Chicago The content was codesigned by a local review board along with community imams, who would then deliver the health messaging in the form of sermons. The sermons were expressly tailored towards Muslim American women, in USA	235 Muslim participants, age (18 +), predominately male (South-Asian, Arab, White)	Two 30 to 45-min sermons were presented to encourage better health in American Muslims	A questionnaire consisting of information about demographic data, perceived acceptance of the program, and participant knowledge	Most of the content was seen to be appropriate with participants willing to engage in subsequent health-related lectures. No substantial differences in tolerability of sermon or sermon-provide by either race, gender, or ethnicity
Islam et al. (2018) explored the outcomes aimed at enhancing the management of those with type -2 diabetes among New York City Bangladeshi people	Community centres, USA. Program facilitated by bilingual local health professionals with a specific focus on the Bangladeshi community in New York City, USA	336 Muslim participants (176 treatment group, 160 control group). Bangladeshi, age (21–75), residing in metropolitan areas, with diabetes type 2 diagnosis	Educational seminar, which providing a summary of diabetes type 2 The following four meetings incorporated group instruction on nutritious food, the significance of and approaches for boosting physical movement, possible problems diabetes type 2 and preventative self-care, and stress managing and family assistance	A screening survey for demographic data. Glycaemic control, and other physiological measures	Improvement in diastolic and systolic blood pressure, cholesterol, triglycerides, BMI, weight, blood pressure, and knowledge at 6 month and 1 year post-intervention in treatment group, no changes in control group
Abdulwasi et al. (2018) examined issues affecting Asian Muslim women’s willingness to engage in physical tasks within the mosque setting	This mosque-based initiative looked at ways to increase physical activity amongst Muslim women in Ontario, Canada	12 Muslim female participants (South-Asian), age (23–74), 50% employed, 50% unemployed. Income < 60 K per year. Resided in Canada > 10 years	Diabetes prevention program incorporated aerobic and circuit training to promote increased. physical activity	Individual semi-structured interviews	Attendance of a professional trainer (83%). 75% of participants reported intervention to be appreciated and convenient. Participants reported persistent participation in the exercise program due to feeling supported by their husbands and instructors. Majority of participants reported feeling comfortable in the intervention because of its implementation within the mosque
Maynard et al. (2017) examined the enrolment, appraisal, and judgement methods of programs aimed at reducing obesity amongst ethnically diverse children	This program was used to understand contributing factors of obesity among ethnically diverse marginalize children. Delivered at six schools (three primary, three secondary), and places of worship (two churches, two temples and two mosques), in the UK	65 children (physical activity intervention), 155 children (dietary evaluation). Children (Hindu, Muslim, Christian), age (8–13)	One-off program focussed on the development of healthy habits, particularly in relation to diet. The program was delivered within schools, churches, and mosques	Dietary journals, physical activity, and self-efficacy surveys	Although not specifically intended for Muslims, delivering interventions in the mosque can reach a larger population. Completion of intervention was high. Program sessions were assessed amongst 155 children in the five participating schools, 33 children in temples, churches, and mosques. Appraisal coverage was more reliable in these establishments compared with schools
King et al. (2017) explored the viability and willingness to provide Second-hand smoke (SHS) education programs within several mosques across the UK	Religious teachers facilitated the intervention aimed at raising awareness of Second-hand Smoke (SHS) inhalation among Bangladeshi and Pakistani Muslims in the UK	7 participants and a group of women, a group of children, and a group of men	Smoke Free Homes (SFH) focussed on providing information on the dangers of second-hand smoke in the form of leaflets and delivered via sermons	Individual interview and focus group discussions	Participants decided that Religious Teachers were appropriate instructors of health information. A variety of religious establishments in terms of the comparative sizes of worshipers and mosque staff, locality, and structure. Participants particularly cited refraining from smoking around children, and only smoking outside the home
Banerjee et al. (2017) assessed the appropriateness and efficacy of providing Asian Muslim women with physical activity outlets within mosques in Canada	The program was delivered by physiotherapists and kinesiologists and supervised by a nurse practitioner. The physical activity intervention was carefully crafted for South Asian Muslim women in Canada	62 female Muslim participants (South-Asian: India, Sri-Lanka, Pakistan, Bangladesh), age (18 +), member of mosque community	Program provided a series of aerobic exercises and circuit training	Pre–post-questionnaire using Duke Activity Status Index (DASI) and International Physical Activity Questionnaire	Participants showed a considerable rise in median scores of self-efficacies, readiness, and the value of partaking in a physical routine. Most participants positively evaluated the exercise sessions, citing that they were convenient, helpful, informative, and easy to follow
Siddique and Mitchell (2013) sought to understand the differences in attitudes and behaviours towards oral cancer risks between older and younger Gujarati Muslims	General medical practitioners, general dental practitioners, and hospital doctors tailored the initiative for Gujarati Muslims in West Yorkshire, UK. The study setting was in the Community Health Fair involving the Indian Muslim Welfare Association	96 female and male Gujarati Muslim participants, aged between 16 and 81 years	The intervention aimed to deliver health education about oral cancer risk factors in the forms of lectures, workshops by relevant medical experts	Pre- and post-intervention bilingual questionnaires	The assessment showed a very low level of alcohol consumption among Gujarati Muslims. In terms of tobacco consumption, first generation Gujarati men consume more tobacco than second generation. In general, it was found that knowledge about risk factors for oral cancer increased after the intervention. This local community-based health promotion program has proven to be effective in improving health literacy
Marinescu et al. (2013) aimed to design and implement a physical activity program for women who come from backgrounds where co-ed exercise is forbidden	A program was facilitated by local health care workers to encourage increase physical activity amongst women in low-income marginalized groups within the public housing sectors of Seattle, USA. The study setting was in the community centres	239 participants were recruited from two low-income public housing communities. The majority of participants were from East – African (Somali)	Women only physical exercise classes and 10 free swimming sessions in order to increase physical activity amongst women	A community impact evaluation: An interview-based survey (in participants’ first language). Accelerometer measurement to measure physical activity time	Women articulated the prominence of physical exercise; however, their lifestyle changes have caused them to be inactive. Women appreciated the opportunity to exercise in a women-only environment, in which they felt comfortable and safe. Of the 134 women-only sports classes, the average attendance was 9.8 participants per class, and each participant attended an average of 6.7 classes. Participants registered and prepaid for 10 weekly swimming classes per season
Islam et al. (2012) described the findings of an initiative to enhance diabetes intervention programs for New York City Bangladeshi health care personnel	Bangladeshi Muslims in New York City, USA participated in a health care worker initiative. The study setting was in the clinic and community centre	47 Bangladeshi participants (men and women). Low English proficiency Average income < 25 K	NYC Bangladeshi participants formed focus groups to explore health-related attitudes and behaviours in relation to the prevention and management of diabetes Organizers combined the notion of *niyom* [rules and routines of life] to encourage engagement in healthy lifestyles choices	72 item self-report questionnaire and focus group discussions	Baseline survey reported that 26% of participants had diabetes. Majority of participants did not understand the meaning of Haemoglobin A1C. Most diabetic participants were using medications to control their blood glucose. Qualitative findings elaborated factors influencing diabetes control, i.e. psychosocial, cultural-religious, and structural-environmental factors
Grace et al. (2008) explored the opinions and positions of the Bangladeshi people in relation to diabetes prevention strategies	Religious officials, Islamic academics, and health professionals ran an intervention within general practices, community centres and mosques to support the low socio-economic Bangladeshi community in the London borough of Tower Hamlets, UK	80 Bangladeshis without diabetes (with or without family history of diabetes), 29 male religious leaders, and 20 health professionals	Stage 1: Bangladeshis’ attitudes, values, and beliefs towards diabetes prevention were assessed Stage 2: Islamic scholars’ attitudes, values, and beliefs towards diabetes prevention were examined Stage 3: Health professionals’ attitudes and experiences working with. Bangladeshi community were explored	Questionnaires and focus group discussions	Most participants accepted the concept of diabetes prevention and were more knowledgeable than expectation. Islamic scholars perceived that faith-based interventions were associated with Muslims’ behaviour change. Health professionals are reluctant to discuss lifestyle changes in consulting, partly because of their poor understanding of their culture and religion as well as stereotypes against Muslims
Tse (2002) Provided training for bilingual Muslim workers to deliver cultural and gender sensitive assistance for women experiencing depression	Culturally appropriate intervention design in Western Australia Ethnically diverse community workers were trained to offer psychoeducation surrounding depression and postnatal depression for Muslim women	20 community workers [16 successfully completed their training] aged 31 to 51 years, homemakers, from 11 different ethnicities, had lived in Australia for < 10 years and been involved in community activities for > 5 years	Program was designed to train Muslim community workers who were in a crucial position to provide cultural and gender appropriate support. It consisted of six components aimed at coping with depression	Questionnaire using 17 item 4-point Likert scale to evaluate the module content, relevancy, and teaching methods; and an analogue scale to assess participants’ interest level pre- and post-intervention	Participants reported a high satisfactory level of the module content and delivery. They had greater interest after completing the modules. Participants thought that their involvement in the program increased their cross-cultural understanding. Participants were able to apply the new knowledge and skills to provide appropriate support to women with depression or postnatal depression

### System Characteristics

System characteristics include the country of origin of the studies, types of organizations, whether mosque- or community-based, and staffing for the interventions. Nine studies were conducted in USA, five in UK, four in Canada, and one in Australia. Half of the studies used mosque-based educational interventions, 25% were conducted in community centres, and the remaining used a variety of places (i.e. combination of: mosques and either community centres or schools; mosques, clinics, and community centres). One study from Australia (Tse, 2002) did not specify the setting used for delivery of a training for sixteen community workers. In addition, a wide range of skills and expertise was involved in designing, delivering educational programmes, and evaluating health and social care outcomes. The mixture of organizing groups, inclusive of health and non-health professionals, was in most studies reviewed. Some studies did not mention health workers or relevant stakeholder involvement in design, delivery, and evaluation stages of the interventions (Abdulwasi et al., 2018; King et al., 2017; Vu et al., 2018).

Health promotion programmes in this review were developed by delegates from several research teams, social service associations, community centres, and community mosques, focussing on Muslim-American women from various community groups (Padela et al., 2018; Padela, Malik, Vu, et al., 2018; Padela et al., 2019). In two studies, programmes were facilitated by local health professionals with a specific focus on the Bangladeshi community in New York City (Islam et al., 2012, 2018). Medical doctors and nurses led a programme targeted towards the local Syrian refugee community in Baltimore (Chaudhary et al., 2019). The target population of (Zoellner et al., 2018) consisted of Somali Muslim refugees in a large city in the USA. Muslim women’s views were studied to inform the delivery of effective health messaging in Chicago (Vu et al., 2018). Finally, one health promotion initiative looked at ways to increase physical activity amongst South Asian Muslim women in Ontario (Abdulwasi et al., 2018).

Focused on health promotion, students and parents from multiple primary and secondary schools and faith organizations for Christians, Muslims, Hindus in London, UK, were involved in physical activity facilitated by a professional dancer at the schools (Maynard et al., 2017). Banerjee et al. (2017) also reported on a physical activity intervention, which was carefully crafted for South Asian Muslim women in Canada and delivered by physiotherapists and kinesiologists, supervised by a nurse practitioner. In a low-income, public housing sector of Seattle, USA, interventions facilitated by bilingual local health care workers sought to increase physical activity amongst women (Marinescu et al., 2013). Religious teachers facilitated an intervention to raise awareness of second-hand smoke inhalation among Bangladeshi and Pakistani Muslims (King et al., 2017). General medical practitioners, general dental practitioners, and hospital doctors tailored health promotion for Gujarati Muslims in West Yorkshire, UK, on tobacco consumption and oral cancer (Siddique & Mitchell, 2013).

To ensure cultural homogeneity, programmes were frequently delivered by Muslim facilitators who were either the same gender, ethnicity or spoke the same language as participants. In the Australian study, ethnically diverse Muslim community workers were employed to deliver psychoeducation surrounding depression and postnatal depression to ethnically diverse Muslim women (Tse, 2002). Alternatively, Darko et al. (2020) brought together a mix of Muslim primary health care workers and trained them in delivering a culturally tailored diabetes management programmes to culturally diverse Muslim communities.

### Participant Characteristics

While samples were selected purposively, all studies reviewed had Muslim people as participants, predominantly adults. Population samples ranged from 8 to 2446 participants, the Muslim participants’ representation in the sample ranging from 16 to 100%. Health promotion programmes specifically designed for refugees were the focus of two studies (Chaudhary et al., 2019; Zoellner et al., 2018). Only one study (Maynard et al., 2017) used a mixed sample of Muslims and non-Muslims, including children aged 8 to 13 and their parents. The most commonly discussed issues were women's health, with nine studies focussed on women and specific programmes related to women’s general health and physical activities, breast cancer screening, and depression (Abdulwasi et al., 2018; Banerjee et al., 2017; Marinescu et al., 2013; Padela, Malik, Ally Syeda, et al., 2018; Padela, Malik, Vu, et al., 2018; Padela et al., 2019; Tse, 2002; Vu et al., 2018). Related to individual lifestyle behaviours, the second most common health issue was diabetes as reported in five studies (Darko et al., 2020; Grace et al., 2008; Islam et al., 2012, 2018; Padela, Malik, Vu, et al., 2018), followed by breast cancer screening and awareness (Padela, Malik, Ally Syeda, et al., 2018; Padela, Malik, Vu, et al., 2018; Padela et al., 2019). One study focused on lifestyle and obesity related illness in children (Maynard et al., 2017). Health promotion content specific to smoking and oral cancer was the focus of two studies (King et al., 2017; Siddique & Mitchell, 2013). Apart from women-specific depression and suicide prevention program, other mental health-related issues, such as addiction and PTSD were also deliberated (Chaudhary et al., 2019; Hassan et al., 2021; Zoellner et al., 2018). While most of these studies used samples of lay people and Imams as the target of interventions, three study samples also included health professionals or community workers (Darko et al., 2020; Grace et al., 2008; Tse, 2002).

### Intervention Methods

Resonance between Islamic teachings, and health and social care messages were frequently cited in most of the studies reviewed. Four studies held central the inherent interconnection of religion and culture for Muslims (King et al., 2017; Vu et al., 2018). King et al. (2017) reported the need to situate programmes within an Islamic context, specifically consideration of Quran, Sunnah and Islamic jurisprudence when developing interventions with Muslims. Islam et al. (2012) identified Muslims as having a religious commitment to build healthy habits and look after themselves, hence integrated ethnoreligious values and norms in their psychoeducational design. Islamic components considered important to achieving effective outcomes included people and place, i.e. mosque-based interventions, Imam’s presence, use of the Quran, and Sunnah to implement their programmes in a cultural manner (Chaudhary et al., 2019; Padela et al., 2018; Vu et al., 2018). Opportunities to break for prayer or using religiously tailored messages were reported as key to contribute to the success of interventions (Marinescu et al., 2013; Padela et al., 2019).

Ethnoreligious adaptation of interventions originally designed for non-Muslim populations was an important consideration in the delivery of interventions, such as those delivered at community centres and mosques for a Bangladeshi community in a London borough (Grace et al., 2008). Content in the interventions was generally co-created and delivered by individuals perceived to have cultural alliance or competence. In doing so, two major concerns were addressed: (1) that programmes were reviewed, delivered by Imams, and delivered in Mosques; (2) information and capacity-building activities were complemented with the Quran and Sunnah informed messages (Grace et al., 2008; Hassan et al., 2021; Padela et al., 2019; Zoellner et al., 2018). The use of the Quran and prophetic stories, was repeatedly confirmed in studies like Hassan et al. (2021) and Zoellner et al. (2018). The content, sermon scripts, and health messages were reviewed by the Imams to ensure theological accuracy and validity of the content (Chaudhary et al., 2019). Intervention content for delivery in the broader community was often co-created with Muslim participants in focus group discussions, which aimed to ensure ethnoreligious acceptability (Abdulwasi et al., 2018; King et al., 2017; Padela et al., 2018). Muslim professionals, e.g. general dental practitioners, general medical practitioners, psychiatrists, and health workers, were employed to deliver interventions in schools and community centres (Banerjee et al., 2017; Darko et al., 2020; Islam et al., 2018; Siddique & Mitchell, 2013), whereas delivery of health and social care messages in mosques was done by Imams (King et al., 2017; Padela et al., 2019; Padela, Malik, Vu, et al., 2018). While Imam-led sermons or classes were found effective in promoting women’s health, Vu et al. (2018) argued that Imams should be trained with health-related knowledge and healthcare workers should have religio-cultural competency to ensure intervention effectiveness.

Studies also focused on session structure, duration, and flexibility for Muslims, with some variation across them. Information sermons lasted for 30–45 min (Padela et al., 2018) and short programmes ranged from two to six sessions of two to four hours duration (Padela, Malik, Vu, et al., 2018; Zoellner et al., 2018). The longer programmes involved between five to 15 weekly interventions (Chaudhary et al., 2019; Hassan et al., 2021; Tse, 2002). Flexibility for participation was considered in two interventions, evident in the conscious consideration of pray times and gender-specific approaches (Marinescu et al., 2013; Zoellner et al., 2018).

### Programme Outcomes

Three themes emerged including acceptance of the interventions, improved health and social care literacy, and changes in beliefs and behaviours for achieving health and wellbeing. Ten articles had specific focus on Muslim women’s participation, and associations between socio-cultural variables and intervention acceptance (Abdulwasi et al., 2018; Banerjee et al., 2017; Marinescu et al., 2013; Padela et al., 2018; Padela et al., 2019; Padela, Malik, Vu, et al., 2018; Tse, 2002; Vu et al., 2018). In considering religion as a key socio-cultural variable, acceptance was evidenced in two ways: completion rates and feeling culturally safe.

The study by Maynard et al. (2017) on healthy lifestyles required 24-h diet recalls and self-efficacy questionnaires and generated completion rates ranging from 89 to 100%. Padela et al. (2018) established that health and social care-based sermons were desired by 67% of participants and that the actual sermons were perceived as religiously acceptable by 72%. Banerjee et al. (2017) reported the average number of exercise classes attended by the participants was 20 out of 28, while the average number of women attending each session was 13/28. In terms of feeling culturally safe, one programme on Islamic trauma healing administered a client satisfaction survey (Zoellner et al., 2018). All respondents indicated “excellent” in response to integration of religious beliefs and cultural practices into interventions (poor = 1/excellent = 4; M = 4.00, SD = 0.00). Findings from mixed-methods and qualitative studies confirmed the acceptance of religious tailoring of health promotion messages in Islamic sermons (Abdulwasi et al., 2018; King et al., 2017; Marinescu et al., 2013). It is important to note that there were no significant differences in acceptability when comparing sermon or sermon-giver, by gender or race/ethnicity.

Both Muslim women and men reported increased awareness of health and social protective mechanisms when religious and socio-cultural elements were embedded in the interventions (Islam et al., 2012; Siddique & Mitchell, 2013). Siddique and Mitchell (2013), for example, showed that religious tailoring of health promotion improved health literacy postvention; evidenced by improved identification of oral cancer risk factors compared with baseline (difference 0.40, 95% CI 0.23 to 0.57, *p* =  < 0.001). Substantial improvements in participants’ health and social wellbeing were shown in quantitative and qualitative studies when religious references from the Quran and Sunnah were included and delivered by Imams, both in self-reported knowledge (Chaudhary et al., 2019; Hassan et al., 2021) and in observed application of new knowledge in self-care (Padela, Malik, Vu, et al., 2018; Tse, 2002; Vu et al., 2018). In terms of health and social care, religious tailored interventions also reduced detrimental health beliefs and adverse behaviours. For example, one study noted a significant decrease in agreement with the belief, ‘Breast Cancer Screening is not important because Allah decides who will get cancer’ (− 0.40, *p* = 0.03) (Padela et al., 2019). The use of religious messages to counter such health beliefs, such as this, was perceived across the studies as critical to change.

Religious beliefs shaped many of the Muslim participants’ beliefs and behaviours, with most studies confirming that religious tailoring improved the impact of interventions. Increased and sustained willingness to engage with health and social care following interventions, compared to baseline, were reported in two studies (Hassan et al., 2021; Padela, Malik, Ally Syeda, et al., 2018). Other studies noted changes associated with religious tailoring that included increases in readiness, self-efficacy, and refraining from engaging in adverse behaviours in the presence of children (Banerjee et al., 2017; King et al., 2017). Integrating scriptural references one study resulted in an improvement in health behaviour (Padela et al., 2018). The integration of ethnoreligious elements, including Quranic references, gender-specific programmes, delivered in familiar languages, reportedly improved engagement among Muslims and better health and social care outcomes overall.

## Discussion

Ethnicity and religion, as part of the broader socio-cultural system, impacts health behaviours and experiences of wellbeing (Kawachi, 2020). International researchers and world health institutions recognize how Islamic faith interacts with health beliefs, health and social care outcomes (Alomair et al., 2021; Shahin et al., 2021; Stroope et al., 2019). For example, the Amman Declaration affirms 60 Islamic teachings about healthy and harmful behaviours, which are important for physical, mental and social wellbeing (WHO, 1996). Muslims hold the messages of Islamic Classical texts, including from the Quran, as way of life. Such texts explain that the source of human peace and comfort comes from following Islamic guidelines (Attum et al., 2021).

A holistic biopsychosocial approach to health must incorporate religion for Muslims, where religion is integral to their overall wellbeing. Adaptations of services are an important implementation strategy (Miller et al., 2020). Religious considerations when designing programmes and interventions ensures a holistic biosocial approach in the care of Muslim-minority populations (Attum et al., 2021; Irajpur & Moghimiyan, 2018). When designed in accordance with Islamic teachings, the studies we reviewed indicated considerable potential to improve intervention outcomes. However, this required a wide range of skills and expertise, from health and non-health professionals to local religious authorities.

Most of the studies we reviewed focused on Muslim-minority populations in the USA, UK, and Canada, where there have been rapid changes in migration, population patterns, culture, and religion. In applying the QHOM to understand socio-cultural influences, we noted the importance of religious integration in health and social care policy, programmes, and messages when engaging with these populations. Two studies (Vu et al., 2018; Zoellner et al., 2018) provided strong evidence in the importance of interdisciplinary and interorganizational collaborations when co-designing programme materials. Imams were perceived as trusted messengers among the Muslim communities and their engagement in co-design was found necessary to leverage community engagement and change.

In the studies reviewed, researchers and practitioners allied with Imams to elaborate capacity to extend health and social care messages for Muslim-minorities experiencing health and social disparities. Consistent with broader understandings, Imams are regarded as key advisors who endorse Muslims’ health and social behaviour changes in accordance with Islamic principles (Cohen-Dar & Obeid, 2017; Usman & Iskandar, 2021). While Imams were identified as important research collaborators, there were some difficulties identified in this review due to different knowledge expertise brought by each of the religious leaders and researchers. In addition to religious tailoring and co-design, bilingual professionals or community workers were crucial for culturally appropriate communications and monitoring of intervention outcomes.

It is generally agreed that religious-based health and social care programmes in the community, with minority groups, are more likely to contribute to better health and social wellbeing outcomes (Alomair et al., 2021; Shahin et al., 2021; Stroope et al., 2019). This is because religion influences values, knowledge, behaviours and understanding of health and wellbeing. When many Muslims judge Quranic messages as important for coping, and that following religious guidelines will result in peace and comfort (Attum et al., 2021), then the positive impact of religion on health and wellbeing seems logical. While some studies reported little or no association between religiosity, health status and adjustment to illness (Fitchett et al., 1999; Irajpur & Moghimiyan, 2018; Page et al., 2020), overall our review indicated positive outcomes from religious tailoring due to identified associations between cultural relevance and the greater likelihood of sustained health behaviour change.

The studies in our sample more likely reported effective outcomes when religiously tailored interventions focused on health behaviours related to tobacco use, physical activity, alcohol and drug use, or lifestyle diseases such as cardiovascular disease and diabetes. Even though interpretations of religion, ethnicity and culture were diverse, the commitment to Allah in all aspects of life were drawn upon to deliver health and social care concepts collectively to Muslim people in dissemination of health messages. The heightened need for connectedness to the Quran and Sunnah, the need for spiritual peace, and the need for comfort among Muslim-minorities, is likely to make religiously tailored health messages more effective. This is consistent with findings of a comprehensive review by Abuelezam et al. (2018) of 247 studies on Arab Americans’ health behaviours and health outcomes, through religiously tailored messages that significantly altered the people’s preventative health-related behaviours.

Our review found that women’s health received scrupulous interest from the researchers. This was with consideration of Islam’s emphasis on health as a holistic state of physical, psychological, spiritual, and social wellbeing (Attum et al., 2021; Irajpur & Moghimiyan, 2018). Depending on the level of their religiosity, women are influenced by the Quran, Hadith, and Sunnah on healthy living in two ways: first, via direct statements advising what women should do; second, via examples of women as role models to follow (Darzi et al., 2021). The foundations of lifelong health, based on religious messages, therefore rest on holistic emphasis of health promotion and health prevention.

Based on the Islamic perspective, the foundations of holistic wellbeing for a Muslim are believed to form across four important stages, namely at conception, in the womb, at birth, and during infancy and childhood (Alimohammadi et al., 2020). As a result, religious teachings advise that Muslim women must maintain excellent physical, psychological, spiritual, and social health, especially during pregnancy, so that healthy offspring are born. While obeying religious guidelines may not be seen as an absolute requirement for good health, these principles provide a basis and guidance for being a healthy Muslim. In consideration, Jabbari et al. (2020), showed that listening to Quran recitation with or without translation during pregnancy significantly reduced perceived stress, anxiety, and depression levels in Iranian pregnant women. Participating in an act of worship, gave the women a feeling of being closer to piety and thus gave peace of mind and provided a source of strength and spirituality. In another study by Komariah et al. (2020), focused on Muslim women undergoing chemotherapy, they showed that Islamic-based care improved spiritual wellbeing, reduced anxiety and improved coping. This evidence brings to light the relevance of the Islamic principles that have significant impacts on women’s health beliefs, behaviours, and overall wellbeing.

Many of the studies included reported high intervention uptake rates among Muslims, arguably due to having appropriate religio-cultural tailoring in their designs. Acceptance was measured by programme completions as opposed to application of any acceptance measurement scale or measurement of behaviour change. According to Ajzen (1985) in his Theory of Planned Behaviour, Muslims’ behaviours can be understood via the principle of compatibility. This helps to explain how attitudes of Muslim women towards mammograms, for instance, and their actions in having mammograms need to occur without conflict. Therefore, if interventions are contextualized in accordance to religious and cultural values; acceptance, actions, and behaviour change (although not the only determining variables) are more likely.

Change outcomes depend on a range of factors that may not necessarily be under the control of individuals, such as availability of resources and opportunities to display the behaviours (Ajzen, 2005). Ajzen (2005) explained Muslim’s tendency to apply religious evaluation in weighing up positives and negatives influence attitudes towards a given behaviour. As well, there are normative beliefs formed around Muslims’ support systems, which will influence subjective norms and individual decision making. Past experiences, such as whether Muslims have ever engaged in a particular behaviour, will influence perceived control over circumstances and influence change outcomes accordingly. These were observed in one of the studies in our review, in which Padela, Malik, Vu, et al. (2018) explored how Muslim women’s behavioural beliefs, perceptions of pain and fear, normative beliefs on cultural taboos and a ‘women only’ issues, and control beliefs (i.e. Allah’s Will) may prevent women from undergoing mammograms. These distinctive beliefs provide insight into the underlying complex thought processes of human behaviour, including the role of Islamic beliefs that add to the multifaceted complexity of cognitive processes in the construction of Muslim behaviours.

Health and social care interventions can no longer be identified in terms of individual capacity to search, comprehend, and act on new information, but also the emphasizes the capacity of health information providers. The ‘Health People 2030’ defines organizational literacy as the capacity of organizations follow policies that equitably enable individuals to access information pertinent to improving, including health and social care professionals, health and wellbeing (Brach & Harris, 2021). Interventions to improve Muslim’s overall health and social wellbeing are unlikely to be effective when organizations or care providers do not deliver interventions in accordance with Islamic beliefs. For Muslims, health is a holistic state of physical, psychological, spiritual, and social wellbeing and is believed to be the greatest blessing Allah has given to humans. Islam attaches significant importance to health, so taking care of one’s health is a religious duty. Intervention programmes designed in conjunction with Islamic values, therefore, can effectively improve Muslims’ overall health.

Higher levels of health and social care literacy have associations with better compliance with interventions and sustained outcomes (Netemeyer et al., 2020). For example, the study by Elkalmi et al. (2021) recently provided insight into how health promotion information incorporating ‘Halal’ and ‘Haram’ impacted Muslims’ attitudes towards vaccination programmes. Vazifeh doust et al. (2020) demonstrated how Islamic-based treatments in hospital settings were effective in helping children with cancer to adapt to treatment. Ahaddour et al. (2020) showed how Islamic beliefs integrated into aged care facilities in Belgium provided older Muslim residents with a sense of dignity. These examples provide a seamless illustration depicting the harmony of personal and organizational health literacy, through respecting that Islamic beliefs are essential to a Muslim’s way of life, in childhood, throughout life and in death.

## Limitations

Our initial interest in undertaking this review was to generate evidence to inform the development of health and social care interventions in Australia, as part of a Muslim and non-Muslim health promotion and social care partnership. While our Muslim partners advised of the importance of Mosque-based and Muslim community interventions to the people in their communities, a limitation of this review study is the small quantity of research that could be located via rigorous searching for relevant articles.

We extended our review from our initial focus on Australian Mosque-based interventions to Mosque- and Muslim-community-based interventions in Australia and other Anglophone countries, such as Canada, UK, and the USA. There were still relatively few studies located. We suggest that the limitation of this study is not necessarily due to any methodological insufficiency. Instead, we have learned through our Muslim and non-Muslim partnership, that Mosques may be perceived in Anglophone countries as places of worship as opposed to also being community services requiring funding to develop Mosque-based services in support of these communities-funding which is not often available. Concomitantly there is limited research about Mosque-based interventions when the interventions themselves are few. This is opposed to the unquestioned funding in Australia and other Anglophone counties in favour of Church-based and Christian community health and welfare interventions.

As a result, this study has both strengths and limitations. A particular strength of our integrative review was to summarize a combination of research utilizing diverse methodologies, systematically and rigorously. We have provided a comprehensive understanding of Muslim-minority health and social care interventions delivered in Mosque and Muslim community settings in non-Muslim countries, and the intervention outcomes in an emerging and also important field of practice in a rapidly evolving multicultural and religiously fluid world.

## Conclusion

Evaluating religiosity as it relates to health and socio-cultural care provides insight into the meaningfulness of experiences associated with Islamic principles and resources, such as Quran and Sunnah (i.e. Prophetic stories). Religious and cultural care in the studies reviewed included focus on spirituality, faith, geographical locations, kinship, and ethnicity. While all aspects are important, intervening in ways that matter to Muslims was reported as likely to influence change in health beliefs, social care service engagement and health and wellbeing behaviours. Hence, health in Islamic terms must extend to physical, psychological, spiritual, and social aspects of health, social care, and wellbeing, to optimize intervention outcomes.

Capturing how religious involvement relates to interventions and health behaviour change in Islamic societies is a complex matter. Across the studies from Australia, Canada, UK, and USA, interventions were formulated with appreciation of religious and cultural principles. Each showed some level of effectiveness associated with religious tailoring and adaptations to programmes, but conclusions were generally not strong. Studies did not measure or consider participants' levels of religiosity, which is an important confounding variable in which to understand intervention effect. Consequently, it is still now known whether religiously tailored interventions with Muslims caused behaviour changes, in the programmes studied, any differently to non-religiously tailored interventions with Muslims. While certain favourable outcomes could be identified, our interpretations were limited by the strength and quality of evidence reported. In consideration that health and social care is a basic human right, further research to inform policy and practice advocacy is critically important when non-Muslim societies that have become increasingly diverse in religion and culture. This is needed to understand how religious tailoring, for whom and in what ways, offers the greatest benefit to health and social care.

### Supplementary Information

Below is the link to the electronic supplementary material.

Supplementary file1 (DOCX 31 kb)

## References

[CR1] Abdulwasi M, Bhardwaj M, Nakamura Y, Zawi M, Price J, Harvey P, Banerjee AT (2018). An ecological exploration of facilitators to participation in a mosque-based physical activity program for South Asian Muslim Women. Journal of Physical Activity & Health.

[CR2] Abuelezam NN, El-Sayed AM, Galea S (2018). The health of Arab Americans in the United States: An updated comprehensive literature review. Frontiers in Public Health.

[CR3] Ahaddour C, Van den Branden S, Broeckaert B (2020). “What Goes Around Comes Around”: Attitudes and practices regarding ageing and care for the elderly among Moroccan Muslim women living in Antwerp (Belgium). Journal of Religion and Health.

[CR4] Ajzen I, Kuhl J, Beckmann J (1985). From intentions to actions: A theory of planned behavior. Action control: From cognition to behavior.

[CR5] Ajzen I (2005). Attitudes, personality and behaviour.

[CR6] Alimohammadi N, Jafari-Mianaei S, Bankipoor-Fard A-H, Hasanpour M (2020). Laying the foundations of lifelong health at the beginning of life: Islamic perspective. Journal of Religion and Health.

[CR7] Allen C (2021). Contemporary experiences of Islamophobia in today’s United Kingdom: Findings from ten small-scale studies. Insight Turkey.

[CR8] Alomair N, Alageel S, Davies N, Bailey JV (2021). Sexual and reproductive health knowledge, perceptions and experiences of women in Saudi Arabia: A qualitative study. Ethnicity & Health.

[CR9] Attum, B., Hafiz, S., Malik, A., & Shamoon, Z. (2021). Cultural competence in the care of Muslim patients and their families. In *StatPearls [Internet]*. StatPearls Publishing. https://www.ncbi.nlm.nih.gov/books/NBK499933/29763108

[CR10] Bader A, Musshauser D, Sahin F, Bezirkan H, Hochleitner M (2006). The Mosque Campaign: A cardiovascular prevention program for female Turkish immigrants. Wiener Klinische Wochenschrift.

[CR11] Banerjee AT, Landry M, Zawi M, Childerhose D, Stephens N, Shafique A, Price J (2017). A pilot examination of a mosque-based physical activity intervention for South Asian Muslim women in Ontario, Canada. Journal of Immigrant and Minority Health.

[CR12] Bawadi H, Al-Hamdan Z, Ahmad MM (2020). Needs of migrant Arab Muslim childbearing women in the United Kingdom. Journal of Transcultural Nursing.

[CR13] Brach C, Harris LM (2021). Healthy people 2030 health literacy definition tells prganizations: Make information and services easy to find, understand, and use. Journal of General Internal Medicine.

[CR14] Chaudhary A, Dosto N, Hill R, Lehmijoki-Gardner M, Sharp P, Hale WD, Galiatsatos P (2019). Community intervention for Syrian Refugees in Baltimore City: The lay health educator program at a local Mosque [Article]. Journal of Religion and Health.

[CR15] Cohen-Dar M, Obeid S (2017). Islamic religious leaders in Israel as social agents for change on health-related issues. Journal of Religion and Health.

[CR16] Covidence. (2015). *Covidence systematic review software, Veritas Health Innovation, Melbourne, Australia*. http://www.covidence.org

[CR17] Darko N, Dallosso H, Hadjiconstantinou M, Hulley K, Khunti K, Davies M (2020). Qualitative evaluation of A Safer Ramadan, a structured education programme that addresses the safer observance of Ramadan for Muslims with Type 2 diabetes. Diabetes Research and Clinical Practice.

[CR18] Darzi G, Ahmadvand A, Nushi M (2021). Revealing gender discourses in the Qur'an: An integrative, dynamic and complex approach. Hervormde Teologiese Studies.

[CR19] Elkalmi RM, Jamshed SQ, Suhaimi AM (2021). Discrepancies and similarities in attitudes, beliefs, and familiarity with vaccination between religious studies and science students in Malaysia: A comparison study. Journal of Religion and Health.

[CR20] Elkassem S, Csiernik R, Mantulak A, Kayssi G, Hussain Y, Lambert K, Bailey P, Choudhary A (2018). Growing up Muslim: The impact of islamophobia on children in a Canadian community. The Journal of Muslim Mental Health.

[CR21] Erlingsson C, Brysiewicz P (2017). A hands-on guide to doing content analysis. African Journal of Emergency Medicine.

[CR22] Fitchett G, Rybarczyk BD, DeMarco GA, Nicholas JJ (1999). The role of religion in medical rehabilitation outcomes: A longitudinal study. Rehabilitation Psychology.

[CR23] Grace C, Begum R, Subhani S, Kopelman P, Greenhalgh T (2008). Prevention of type 2 diabetes in British Bangladeshis: Qualitative study of community, religious, and professional perspectives. British Medical Journal.

[CR24] Griera M, Martínez-Ariño J, Gonzalez FC, D'Amato G (2016). The accommodation of religious diversity in prisons and hospitals in Spain. Multireligious society: Dealing with religious diversity in theory and practice.

[CR25] Hamdiui N, Marchena E, Stein ML, van Steenbergen JE, Crutzen R, van Keulen HM, Reis R, van den Muijsenbergh METC, Timen A (2021). Decision-making, barriers, and facilitators regarding cervical cancer screening participation among Turkish and Moroccan women in the Netherlands: A focus group study. Ethnicity & Health.

[CR26] Hamiduzzaman, M., Siddiquee, N., McLaren, H., & Tareque, M. I. (2022). The COVID-19 risk perceptions, health precautions, and emergency preparedness in older CALD adults in South Australia: A cross-sectional study. *Infection, Disease & Health*, Article IDH246. 10.1016/j.idh.2022.04.001 10.1016/j.idh.2022.04.001 PMC9015960 35527217

[CR27] Hanrieder T (2017). The public valuation of religion in global health governance: Spiritual health and the faith factor. Contemporary Politics.

[CR28] Hassan AN, Ragheb H, Malick A, Abdullah Z, Ahmad Y, Sunderji N, Islam F (2021). Inspiring Muslim minds: Evaluating a spiritually adapted psycho-educational program on addiction to overcome stigma in Canadian Muslim communities. Community Mental Health Journal.

[CR29] Hong QN, Pluye P, Fàbregues S, Bartlett G, Boardman F, Cargo M, Dagenais P, Gagnon M-P, Griffiths F, Nicolau B (2018). Mixed methods appraisal tool (MMAT), version 2018. Registration of Copyright.

[CR30] Irajpur A, Moghimiyan M (2018). Dimensions of the spiritual needs of Muslim chronic patients: A qualitative study. The Journal of Muslim Mental Health.

[CR31] Ishaq B, Østby L, Johannessen A (2021). Muslim religiosity and health outcomes: A cross-sectional study among Muslims in Norway. SSM - Population Health.

[CR32] Islam NS, Tandon D, Mukherji R, Tanner M, Ghosh K, Alam G, Haq M, Rey MJ, Trinh-Shevrin C (2012). Understanding barriers to and facilitators of diabetes control and prevention in the New York City Bangladeshi Community: A mixed-methods approach. American Journal of Public Health.

[CR33] Islam NS, Wyatt LC, Taher M, Riley L, Tandon SD, Tanner M, Mukherji BR, Trinh-Shevrin C (2018). A culturally tailored community health worker intervention leads to improvement in patient-centered outcomes for immigrant patients with type 2 diabetes. Clinical Diabetes.

[CR34] Jabbari B, Mirghafourvand M, Sehhatie F, Mohammad-Alizadeh-Charandabi S (2020). The effect of holly Quran voice with and without translation on stress, anxiety and depression during pregnancy: A randomized controlled trial. Journal of Religion and Health.

[CR35] Kawachi I (2020). Invited commentary: Religion as a social determinant of health. American Journal of Epidemiology.

[CR36] King R, Warsi S, Amos A, Shah S, Mir G, Sheikh A, Siddiqi K (2017). Involving mosques in health promotion programmes: A qualitative exploration of the MCLASS intervention on smoking in the home. Health Education Research.

[CR37] Komariah M, Hatthakit U, Boonyoung N (2020). Impact of islam-based caring intervention on spiritual well-being in muslim women with breast cancer undergoing chemotherapy. Religions.

[CR38] Marinescu LG, Sharify D, Krieger J, Saelens BE, Calleja J, Aden A (2013). Be active together: Supporting physical activity in public housing communities through women-only programs. Progress in Community Health Partnerships-Research Education and Action.

[CR39] Maynard M, Baker G, Harding S (2017). Exploring childhood obesity prevention among diverse ethnic groups in schools and places of worship: Recruitment, acceptability and feasibility of data collection and intervention components. Preventive Medicine Reports.

[CR40] McLaren HJ, Patil TV (2016). Manipulative silences and the politics of representation of boat children in Australian print media. Continuum.

[CR41] McLaren HJ, Patmisari E, Hamiduzzaman M, Jones M, Taylor R (2021). Respect for religiosity: Review of faith integration in health and wellbeing interventions with Muslim minorities. Religions.

[CR42] Miller CJ, Wiltsey-Stirman S, Baumann AA (2020). Iterative decision-making for evaluation of adaptations (IDEA): A decision tree for balancing adaptation, fidelity, and intervention impact. Journal of Community Psychology.

[CR43] Mitchell PH, Ferketich S, Jennings BM (1998). Quality health outcomes model. Image: the Journal of Nursing Scholarship.

[CR44] Netemeyer RG, Dobolyi DG, Abbasi A, Clifford G, Taylor H (2020). Health literacy, health numeracy, and trust in doctor: Effects on key patient health outcomes. The Journal of Consumer Affairs.

[CR45] Padela AI, Malik S, Ahmed N (2018). Acceptability of Friday sermons as a modality for health promotion and education. Journal of Immigrant and Minority Health.

[CR46] Padela AI, Malik S, Ally Syeda A, Quinn M, Hall S, Peek M (2018). Reducing muslim mammography disparities: Outcomes from a religiously tailored mosque-based intervention. Health Education and Behavior.

[CR47] Padela AI, Malik S, Din H, Hall S, Quinn M (2019). Changing Mammography-Related Beliefs Among American Muslim Women: Findings from a religiously-tailored mosque-based intervention. Journal of Immigrant and Minority Health.

[CR48] Padela AI, Malik S, Vu M, Quinn M, Peek M (2018). Developing religiously-tailored health messages for behavioral change: Introducing the reframe, reprioritize, and reform (“3R”) model. Social Science and Medicine.

[CR49] Page MJ, Moher D, Bossuyt PM, Boutron I, Hoffmann TC, Mulrow CD, Shamseer L, Tetzlaff JM, Akl EA, Brennan SE, Chou R, Glanville J, Grimshaw JM, Hróbjartsson A, Lalu MM, Li T, Loder EW, Mayo-Wilson E, McDonald S, McGuinness LA, Stewart LA, Thomas J, Tricco AC, Welch VA, Whiting P, McKenzie JE (2021). PRISMA 2020 explanation and elaboration: Updated guidance and exemplars for reporting systematic reviews. BMJ.

[CR50] Page RL, Peltzer JN, Burdette AM, Hill TD (2020). Religiosity and health: A holistic biopsychosocial perspective. Journal of Holistic Nursing.

[CR51] Patil TV, McLaren HJ (2019). Australian media and Islamophobia: Representations of asylum seeker children. Religions (basel, Switzerland ).

[CR52] Patmisari E, McLaren H, Jones M (2022). Multicultural quality of life predictive effects on wellbeing: A cross-sectional study of a Muslim community in South Australia. Journal of Religion & Spirituality in Social Work: Social Thought.

[CR53] Plaza del Pino FJ, Cala VC, Soriano Ayala E, Dalouh R (2020). Hospitalization experience of Muslim migrants in hospitals in southern Spain-communication, relationship with nurses and culture. A focused ethnography. International Journal of Environmental Research and Public Health.

[CR54] Saidun S, Akhmetova E, Abd Rahman AA (2019). Religious accommodation for Muslim workers and patients in healthcare. Islam and Civilisational Renewal.

[CR55] Samari G (2016). Islamophobia and public health in the United States. American Journal of Public Health.

[CR56] Shahin W, Stupans I, Kennedy G (2021). Health beliefs and chronic illnesses of refugees: A systematic review. Ethnicity & Health.

[CR57] Shlala EH, Jayaweera H (2016). The right to health: Sri Lankan migrant domestic workers in the GCC. Muslim World Journal of Human Rights.

[CR58] Siddique I, Mitchell DA (2013). The impact of a community-based health education programme on oral cancer risk factor awareness among a Gujarati community. British Dental Journal.

[CR59] Silva RL, Oliveira J, Dias C, Pinto IR, Marques JM (2018). How inclusive policies shape prejudice versus acceptance of refugees: A Portuguese study. Peace and Conflict: Journal of Peace Psychology.

[CR60] Stroope S, Kent BV, Zhang Y, Spiegelman D, Kandula NR, Schachter AB, Kanaya A, Shields AE (2019). Mental health and self-rated health among U.S. South Asians: The role of religious group involvement. Ethnicity & Health.

[CR61] Tse T (2002). Islamic community worker training program for the management of depression. The Australian e-Journal for the Advancement of Mental Health.

[CR62] Usman AH, Iskandar A (2021). Analysis of friday sermon duration: Intellectual reflection of classical and contemporary Islamic scholars. Journal of Religious & Theological Information.

[CR63] Vazifeh doust M, Hojjati H, Farhangi H (2020). Effect of spiritual care based on Ghalbe Salim on anxiety in adolescent with cancer. Journal of Religion and Health.

[CR64] Vu M, Muhammad H, Peek ME, Padela AI (2018). Muslim women's perspectives on designing mosque-based women's health interventions-An exploratory qualitative study. Women & Health.

[CR65] WHO. (1996). *Health promotion through Islamic lifestyles: The Amman Declaration*. Regional Office for the Eastern Mediterranean, World Health Organization. https://apps.who.int/iris/handle/10665/119558

[CR66] Worthington Jr, E. L., Hook, J. N., Davis, D. E., Gartner, A. L., & Jennings II, D. J. (2013). Conducting empirical research on religiously accommodative interventions.

[CR67] Zoellner, L., Graham, B., Marks, E., Feeny, N., Bentley, J., Franklin, A., & Lang, D. (2018). Islamic trauma healing: Initial feasibility and pilot data. *Societies*, *8*(3), 47, Article 47. 10.3390/soc8030047

