# Supplementary material for: Health and Social Care Outcomes in the Community: Review of Religious Considerations in Interventions with Muslim-Minorities in Australia, Canada, UK, and the USA
Source: J Relig Health. 2022 Oct 1;63(3):2031–67. doi: 10.1007/s10943-022-01679-2 (PMC11061054; doi:10.1007/s10943-022-01679-2)
Supplement: Supplementary file 1 — Supplementary file1 (DOCX 31 kb) [file 10943_2022_1679_MOESM1_ESM.docx]

**Table S1:** Database and Search Syntax

| **Database** | Searches conducted May 2020 |
| --- | --- |
| Informit (n-41) | ( ((((community OR community-based OR outreach OR mosque-based OR mosque) %4 strateg*)) OR (((community OR community-based OR outreach OR mosque-based OR mosque) %4 prevent*)) OR (((community OR community-based OR outreach OR mosque-based OR mosque) %4 intervention*)) OR (((community OR community-based OR outreach OR mosque-based OR mosque) %4 program*)) OR (((community OR community-based OR outreach OR mosque-based OR mosque) %4 campaign*))) OR (((community OR community-based OR outreach OR mosque-based OR mosque) %4 guide*)) OR (((community OR community-based OR outreach OR mosque-based OR mosque) %4 counsel*))) AND ( (((wellness OR wellbeing OR "well-being" OR "quality of life" OR "life quality" OR "QOL" OR "HRQOL" OR interpersonal OR "inter-personal" OR happiness OR health* OR psychosocial* OR "PSYCHO-SOCIAL" OR "psychosocial" OR lifestyle OR "life-style" OR "physical activity" OR exercise OR diet* OR obesity OR overweight OR smoking OR parenting)) OR (((domestic OR couple* OR spousal OR family OR families OR child* OR parent* OR marital OR marriage* OR intergenerational OR relationship OR social) %4 violent*)) OR (((domestic OR couple* OR spousal OR family OR families OR child* OR parent* OR marital OR marriage* OR intergenerational OR relationship OR social) %4 violence)) OR (((domestic OR couple* OR spousal OR family OR families OR child* OR parent* OR marital OR marriage* OR intergenerational OR relationship OR social) %4 relation*)) OR (((domestic OR couple* OR spousal OR family OR families OR child* OR parent* OR marital OR marriage* OR intergenerational OR relationship OR social) %4 conflict*))) OR (((domestic OR couple* OR spousal OR family OR families OR child* OR parent* OR marital OR marriage* OR intergenerational OR relationship OR social) %4 dynamic*)) OR (((domestic OR couple* OR spousal OR family OR families OR child* OR parent* OR marital OR marriage* OR intergenerational OR relationship OR social) %4 communicat*))) AND ( (Islam* OR Muslim* OR Mohammedan* OR Muhammadan* OR mosque* OR imam*)) |
| ProQuest (n=442) | noft((Islam* OR Muslim* OR Mohammedan* OR Muhammadan* OR mosque* OR imam*)) AND noft(((wellness OR wellbeing OR "well-being" OR "quality of life" OR "life quality" OR QOL OR HRQOL OR interpersonal OR "inter-personal" OR happiness OR health* OR psychosocial* OR "psycho-social" OR "psycho social" OR lifestyle OR "life-style" OR "physical activity" OR exercise OR diet* OR obesity OR overweight OR smoking OR parenting) OR ((domestic OR couple* OR spousal OR family OR families OR child* OR parent* OR marital OR marriage* OR intergenerational OR relationship OR social) NEAR/4 (violent* OR violence OR relation* OR conflict* OR dynamic* OR communicat*)))) AND noft((community OR community-based OR outreach OR mosque-based OR mosque) NEAR/3 (strateg* OR prevent* OR intervention* OR program* OR campaign* OR Guide* OR counsel*)) |
| PsychINFO (n-105) | 1 islam/ or muslims/  2 (islam* or Muslim* or Mohammedan* or Muhammadan* or mosque* or imam*).ti,ab,id.  3 or/1-2  4 health/ or physical health/ or well being/ or physical activity/ or exercise/ or physical fitness/  5 "quality of life"/ or "health related quality of life"/ or lifestyle/ or lifestyle changes/  6 diets/ or obesity/ or overweight/ or tobacco smoking/  7 happiness/  8 parenting/ or interpersonal relationships/ or couples/ or family relations/ or marital relations/ or partners/  9 marital relations/ or marital conflict/ or marital satisfaction/  10 domestic violence/ or battered females/ or child abuse/ or elder abuse/ or emotional abuse/ or exposure to violence/ or family conflict/ or intimate partner violence/ or marital conflict/ or physical abuse/ or sexual abuse/  11 exp parent child communication/ or interpersonal communication/  12 family relations/ or family conflict/ or marriage attitudes/  13 (wellness or wellbeing or "well-being" or "quality of life" or "life quality" or QOL or HRQOL or interpersonal or "inter-personal" or happiness or health* or psychosocial* or "psycho-social" or "psycho social" or lifestyle or "life-style" or "physical activity" or exercise or diet* or obesity or overweight or smoking or parenting or ((domestic or couple* or spousal or family or families or child* or parent* or marital or marriage* or intergenerational or relationship or social) adj5 (violent* or violence or relation* or conflict* or dynamic* or communicat*))).ti,ab,id.  14 or/4-13  15 community services/ or outreach programs/ or support groups/ or social support/  16 community mental health services/  17 ((community or community-based or outreach or mosque-based or mosque) adj5 (strateg* or prevent* or intervention* or program* or campaign* or Guide* or counsel*)).ti,ab,id.  18 or/15-17  19 3 and 14 and 18  20 (editorial or letter).dt.  21 19 not 20 |
| Scopus (n=198) | TITLE-ABS-KEY ( islam* OR muslim* OR mohammedan* OR muhammadan* OR mosque* OR imam* ) AND TITLE-ABS-KEY ( ( ( wellness OR wellbeing OR "well-being" OR "quality off life" OR "life quality" OR QOL OR HRQOL OR interpersonal OR "inter-personal" OR happiness OR health* OR psychosocial* OR "psycho-social" OR "psycho social" OR lifestyle OR "life-style" OR "physical activity" OR exercise OR diet* OR obesity OR overweight OR smoking OR parenting ) OR ( ( domestic OR couple* OR spousal OR family OR families OR child* OR parent* OR marital OR marriage* OR intergenerational OR relationship OR social ) W/4 ( violent* OR violence OR relation* OR conflict* OR dynamic* OR communicat* ) ) ) ) AND TITLE-ABS-KEY ( ( ( community OR "community-based" OR outreach OR "mosque-based" OR mosque ) W/3 ( strateg* OR prevent* OR intervention* OR program* OR campaign* OR guide* OR counsel* ) ) ) |
| Web of Science (n=157) | TS=(Islam* OR Muslim* OR Mohammedan* OR Muhammadan* OR mosque* OR imam*) AND TS=((wellness OR wellbeing OR "well-being" OR "quality of life" OR "life quality" OR QOL OR HRQOL OR interpersonal OR "inter-personal" OR happiness OR health* OR psychosocial* OR "psycho-social" OR "psycho social" OR lifestyle OR "life-style" OR "physical activity" OR exercise OR diet* OR obesity OR overweight OR smoking OR parenting) OR ((domestic OR couple* OR spousal OR family OR families OR child* OR parent* OR marital OR marriage* OR intergenerational OR relationship OR social) NEAR/4 (violent* OR violence OR relation* OR conflict* OR dynamic* OR communicat*))) AND TS=((community OR community-based OR outreach OR mosque-based OR mosque) NEAR/4 (strateg* OR prevent* OR intervention* OR program* OR campaign* OR Guide* OR counsel*)) |
